# FPGLNet: A Lightweight Frequency Prior-Gated Network for EEG Emotion Recognition

**DOI:** 10.3390/s26206464

**Published:** 2026-10-13

**Authors:** Yue Yuan, Yingzhou Bi, Shanrui Liu, Leigang Huo

**Affiliations:** 1 School of Artificial Intelligence, Nanning Normal University, Nanning 530199, China; 2 Guangxi Key Laboratory of Human-Computer Interaction and Intelligent Decision-Making, Nanning Normal University, Nanning 530199, China; 3 College of Computer and Information Engineering, Guangxi Vocational Normal University, Nanning 530199, China

**Keywords:** EEG emotion recognition, lightweight network, frequency domain modeling, dynamic gating

## Abstract

Although deep learning-based electroencephalography (EEG) emotion recognition methods have achieved promising performance, they generally depend on complex network architectures and high computational costs, which limits their deployment in resource-constrained scenarios. Meanwhile, existing approaches mainly focus on local spatiotemporal feature extraction while insufficiently exploiting global frequency-domain dependencies and dynamic information selection during feature propagation, resulting in inadequate spectral information utilization and redundant feature representations. To address these issues, this paper proposes a Frequency Prior-Gated Lightweight Network (FPGLNet) for EEG emotion recognition. First, a Mixed Aggregation Convolution (MAC) module is introduced at the input stage to jointly encode EEG spatiotemporal characteristics through temporal and spatial convolutions. Subsequently, a Fused Fourier Convolution Mixer (FFCM) module is developed to model global frequency-domain dependencies through Fourier transform and integrate multi-scale depthwise convolutions for collaborative frequency-spatial feature representation. Furthermore, a Prior-Gated Feed Forward Network (PGFN) is proposed to dynamically generate prior-guided gating weights based on feature responses, enabling adaptive selection and recalibration of discriminative features across different brain regions and temporal segments. This mechanism enhances emotion-related representations while suppressing noise and redundant information. Owing to the lightweight architecture design, FPGLNet achieves an effective balance between feature representation capability, model complexity, and computational efficiency. Experimental results demonstrate that FPGLNet achieves classification accuracies of 96.22% and 92.69% on the public SEED-IV and SEED-V datasets, respectively. These results validate the effectiveness of the proposed frequency prior-gated strategy in improving EEG feature representation while maintaining competitive recognition performance and deployment efficiency.

## 1. Introduction

Emotion, as a fundamental component of human cognitive activities, is closely associated with advanced cognitive processes, including decision-making, learning, memory, and social interaction. With the rapid development of artificial intelligence, human–computer interaction, and intelligent healthcare, emotion recognition has gradually become an important research direction at the intersection of neuroscience and artificial intelligence [1,2,3,4,5,6,7]. Among various approaches, EEG-based emotion recognition has attracted increasing attention due to its advantages of objectivity, high temporal resolution, and resistance to subjective concealment. It has demonstrated broad application prospects in intelligent healthcare, assisted diagnosis, driver fatigue monitoring, adaptive education, and brain–computer interfaces.

In recent years, EEG emotion recognition methods have gradually evolved from traditional handcrafted feature-based approaches to deep learning-based end-to-end models, including convolutional neural networks (CNNs), graph neural networks (GNNs), and Transformers [8,9,10,11,12,13]. CNNs can effectively extract local spatiotemporal features; however, their limited receptive fields hinder the modeling of long-range dependencies. GNN-based methods enhance spatial representation by constructing brain-region topology graphs, but they usually rely on predefined static graph structures, making it difficult to characterize dynamic changes in functional connectivity. Transformer-based approaches have been introduced into EEG emotion recognition due to their powerful global modeling capability and have achieved promising performance improvements. Nevertheless, the quadratic computational complexity of the multi-head self-attention mechanism results in high computational costs and memory consumption, limiting their efficient deployment on resource-constrained platforms, such as mobile devices and edge computing terminals. With the increasing applications of EEG-based technologies in wearable devices, emotion-assisted interaction, and mental health monitoring, model deployability has become a critical issue [5,6,7]. Therefore, achieving effective global modeling while reducing computational complexity has emerged as an important research direction in EEG emotion recognition.

Existing studies have attempted to improve EEG feature representation through frequency-domain modeling. Liu J et al. [14] introduced a frequency-domain attention mechanism to adaptively assign weights to different frequency bands. Although such approaches enhance spectral feature representation, they mainly focus on local frequency-band enhancement while neglecting global frequency dependencies and dynamic feature regulation, resulting in redundant feature representations and limiting the discriminative capability and generalization performance of models.

**Figure 1:**
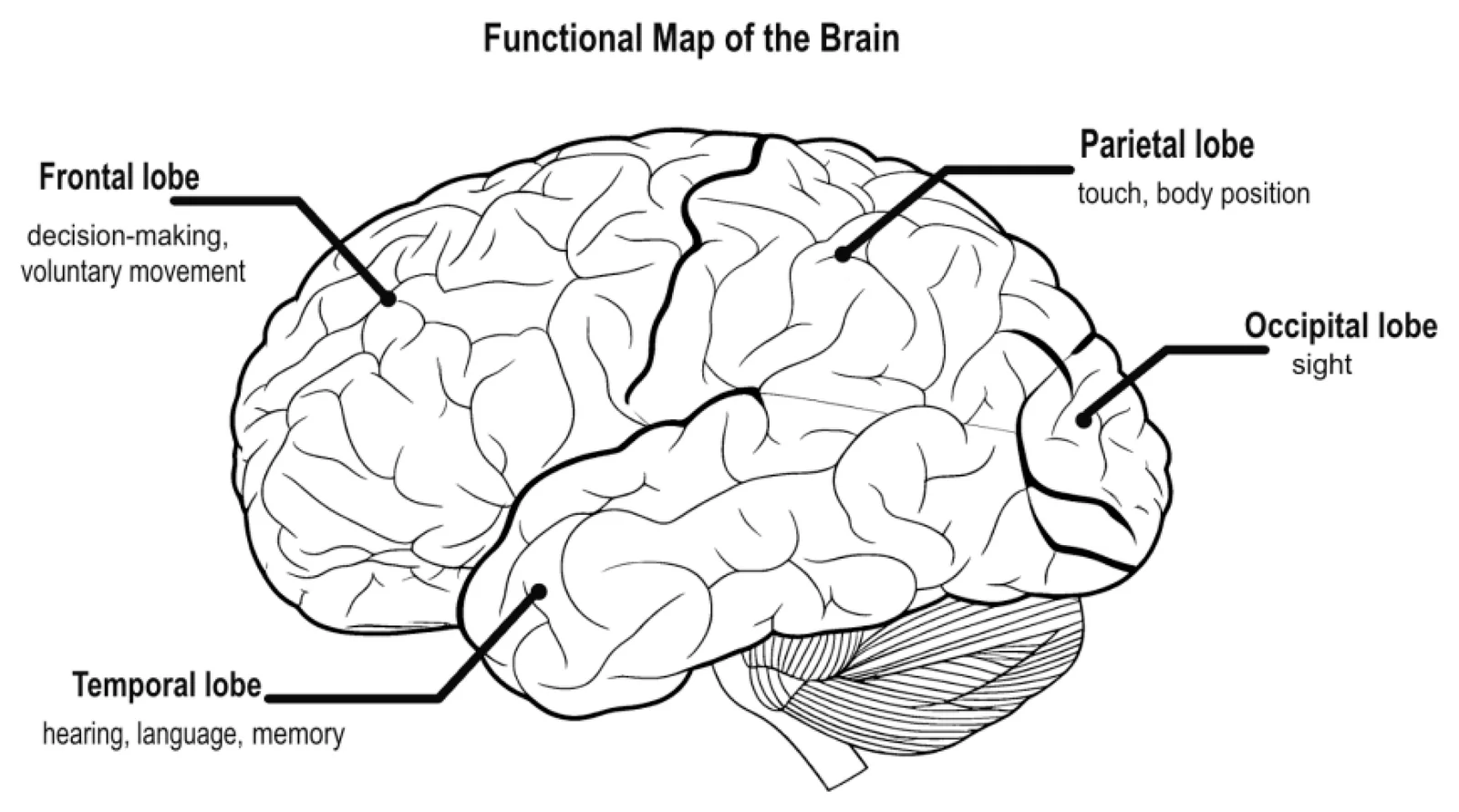
Distribution map of cerebral cortex regions.

Furthermore, as illustrated in Figure 1, emotion-related information is widely distributed across different regions of the cerebral cortex. Among them, the frontal lobe contains abundant emotional information, while interactions among different lobes jointly regulate human behaviors and mental activities [15]. Due to the inherent non-stationarity, multi-dimensional coupling characteristics, and low signal-to-noise ratio of EEG signals, emotion-related information is not only reflected in temporal dynamics but also exists in spatial coordination patterns among different brain regions and spectral distributions across multiple frequency bands. Although current EEG emotion recognition methods have achieved significant progress, most existing models still employ unified linear mapping or feed-forward structures for feature transformation. For example, Liu Z et al. [16] proposed the Swin Transformer, which utilizes a fixed-parameter feed-forward network for feature mapping. Such approaches treat all channels, spatial positions, and temporal segments equally, making it difficult to dynamically adjust information flow according to the discriminative characteristics of individual samples.

To address the aforementioned challenges, this paper proposes a Frequency Prior-Gated Lightweight Network (FPGLNet) for EEG emotion recognition. The proposed network focuses on two key aspects: frequency-domain modeling and prior-guided feature enhancement. First, a Fused Fourier Convolution Mixer (FFCM) module is introduced to integrate Fourier-based frequency-domain modeling with local convolutional feature extraction, establishing collaborative representations between frequency and spatial domains. This design effectively enhances global frequency dependencies and local spatiotemporal feature representation of EEG signals. Furthermore, a Prior-Gated Feed Forward Network (PGFN) is proposed to generate dynamic gating weights based on residual contextual priors, enabling adaptive recalibration of important features from different brain regions and temporal segments. By enhancing emotion-discriminative information and suppressing noisy and redundant features, PGFN further improves the discriminative capability of the model. The entire network adopts a lightweight architecture design. Experimental results on the SEED-IV and SEED-V datasets demonstrate that FPGLNet effectively reduces model parameters and computational complexity while maintaining strong feature representation capability, making it more suitable for EEG emotion recognition tasks in resource-constrained environments. The main contributions of this paper are summarized as follows:

1. A lightweight EEG emotion recognition network, FPGLNet, is proposed. By maintaining a low number of parameters and computational complexity, FPGLNet achieves efficient EEG emotion feature modeling and obtains superior classification performance.
2. A Fused Fourier Convolution Mixer (FFCM) module is introduced. By integrating multi-scale local convolutions with Fourier-based frequency-domain modeling, FFCM enables collaborative modeling of local temporal patterns and global spectral dependencies, thereby enhancing the frequency-domain representation capability of EEG signals.
3. A Prior-Gated Feed Forward Network (PGFN) is proposed. By generating dynamic gating weights based on residual contextual priors, PGFN adaptively selects and recalibrates informative features, effectively strengthening emotion-related EEG representations while suppressing noise and redundant information, thereby improving the discriminative capability of the proposed model.

## 2. Related Work

### 2.1. EEG Emotion Recognition Based on Frequency-Domain Features

EEG signals exhibit prominent multi-band characteristics, where different frequency bands correspond to distinct neural activities and are closely associated with emotional states [17]. Therefore, frequency-domain analysis has gradually become an important research direction in EEG emotion recognition. Existing studies commonly employ techniques such as fast Fourier transform (FFT), power spectral density (PSD), and wavelet transform (WT) to extract spectral features from EEG signals, followed by deep learning models for emotion classification. In recent years, several studies have further introduced attention mechanisms to adaptively model the importance of different frequency bands, thereby enhancing the representation capability of critical spectral information.

### 2.2. Edge Device Deployment in EEG Emotion Recognition

With the rapid development of brain–computer interfaces, wearable intelligent devices, and mobile health monitoring systems, EEG emotion recognition models are increasingly required to be deployed on edge devices, which imposes higher demands on real-time inference, computational efficiency, and memory consumption [18,19]. However, conventional deep neural networks typically involve large numbers of parameters and high computational complexity, making them unsuitable for real-time inference on resource-constrained devices. Consequently, model compression techniques, including knowledge distillation, network pruning, parameter quantization, and lightweight architecture design, have been widely investigated to reduce computational overhead while preserving recognition performance [20,21]. Although these approaches can effectively reduce model complexity, most existing studies mainly focus on structural compression, while paying limited attention to the dynamic preservation of discriminative information and suppression of redundant features during feature propagation. Therefore, further optimization is still required for complex EEG emotion recognition tasks.

## 3. Method

### 3.1. Model Architecture

**Figure 2:**
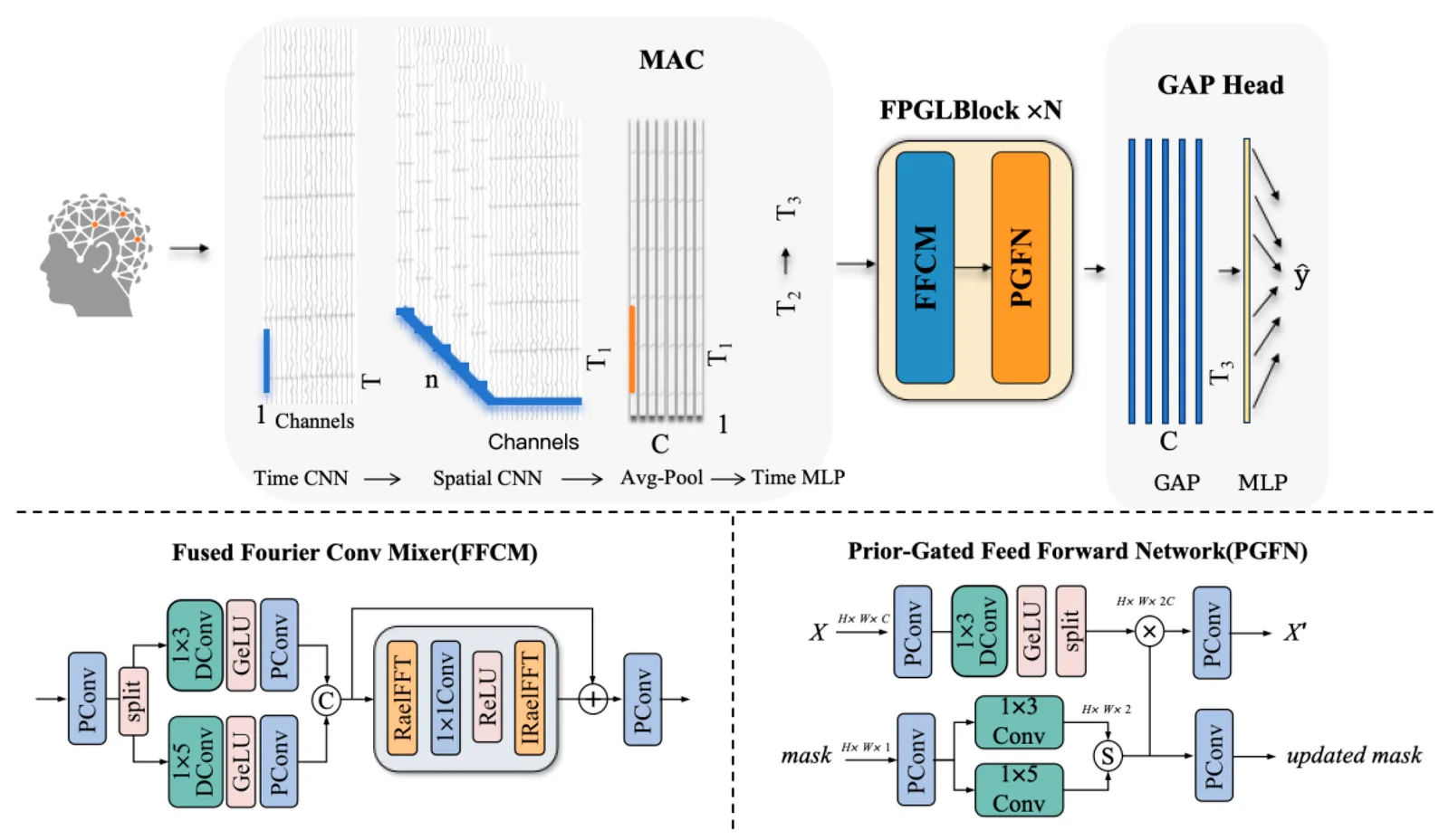
Overall framework diagram of FPGLNet model.

The overall architecture of the proposed network is illustrated in Figure 2. In this work, a novel EEG emotion recognition network is developed by integrating spatiotemporal modeling, frequency-domain enhancement, and prior-guided gating mechanisms. The proposed framework consists of three main components: Mixed Aggregation Convolution (MAC), multiple stacked FPGLBlocks, and a classification head (GAP Head).

First, the MAC module is employed to extract temporal dynamic patterns and spatial topological representations from the raw EEG signals, generating initial feature embeddings. Subsequently, multiple FPGLBlocks are stacked to progressively refine the extracted features. Each FPGLBlock consists of a Fused Fourier Convolution Mixer (FFCM) and a Prior-Gated Feed Forward Network (PGFN). Specifically, FFCM is responsible for local-global feature fusion by jointly modeling frequency-domain dependencies and local spatial representations, while PGFN further enhances discriminative features and suppresses noise through a prior-guided gating mechanism. Finally, the refined representations are fed into the classification head, where global average pooling (GAP) and a multilayer perceptron (MLP) are utilized to predict emotional categories.

The input of the network is the preprocessed EEG signal with a shape of (1, Channels, T), where Channels denotes the number of EEG electrode channels and T represents the number of temporal samples. The output is the predicted class probability vector $\hat{y}$. The detailed architecture of each component in the proposed model is presented in Figure 2.

### 3.2. MAC

Given the input EEG signal denoted as $X\in R^{1\times C_{0}\times T}$ where 1 represents the single input channel dimension, *C*_0_ denotes the number of EEG electrode channels, and *T* indicates the temporal length, the proposed module consists of the following sequential operations.

#### 3.2.1. Temporal Convolution

A convolutional layer with a kernel size of (1, k) and a stride of (1, 1) is first applied to the input signal. This convolution operation is performed exclusively along the temporal dimension to model local dynamic variations within continuous EEG sequences. By covering consecutive k temporal points, the temporal convolution effectively captures local temporal patterns from EEG signals. The temporal convolution process can be formulated as:

(1) $$X_{t}\left(i,j\right)=\sum_{l=0}^{k-1}X\left(1,j,i+1\right)\cdot w\left(l\right)\;$$

The convolution kernel weights are denoted as *W*. After this layer, the output tensor is represented as $X_{t}\in R^{n\times C_{0}\times T_{1}}$, where *n* indicates the number of feature maps extracted by different convolution kernels, and *T*_1_ represents the temporal length after convolution.

#### 3.2.2. Spatial Convolution

To aggregate information from different EEG electrode channels and further establish spatial correlations among distinct brain regions, a convolutional layer with a kernel size of (*C*_0_, 1) and a stride of (1, 1) is applied to *X_t_*. This operation preserves the temporal resolution while integrating information from all electrode channels into a unified feature representation. Therefore, when handling EEG signals with different electrode configurations, the input dimensionality requirements of the proposed framework can be flexibly adapted by simply adjusting the hyperparameter *C*_0_. The spatial convolution operation is formulated as:

(2) $$X_{s}\left(i,t\right)=\sum_{j=0}^{C_{0}-1}X_{t}\left(i,j,t\right)\cdot v\left(j\right)\;$$

where *ν* denotes the spatial convolution kernel weights, *C* represents the number of output channels after spatial aggregation, and the resulting tensor $X_{s}\in R^{C\times 1\times T_{1}}$ represents the fused spatiotemporal feature representation.

#### 3.2.3. Average Pooling Layer

To reduce computational complexity and perform feature smoothing, an average pooling operation is employed to downsample the feature representation along the temporal dimension. Given a pooling kernel size of (1, a) and a stride of (1, t), the pooling operation can be formulated as:

(3) $$X_{p}\left(i,t^{\prime }\right)=\frac{1}{a}\sum_{l=0}^{a-1}X_{s}\left(i,1,t^{\prime }\cdot t+1\right)$$

where the output tensor is denoted as $X_{p}\in R^{C\times 1\times T_{2}}$, and *T*_2_ represents the temporal length after pooling.

#### 3.2.4. Temporal Feature Projection

To enhance the representation capability of temporal features, a nonlinear projection operation is applied to the temporal feature representations. The output tensor can be formulated as:

*Z*(*t*) = *f*(*W*·*X_p_*(*t*) + *b*) (4)

where *W* and *b* denote the learnable weights and bias parameters, respectively, and *f*(⋅) represents the nonlinear activation function. The resulting output tensor is obtained as $Z\in R^{C\times 1\times T_{3}}$.

### 3.3. FPGLBlock

To fully exploit the global frequency-domain dependencies embedded in EEG signals and further enhance feature representation capability, a Frequency-Aware Prior-Gated Learning Block (FPGLBlock) is introduced after the convolutional encoding module and repeatedly stacked to construct deeper feature representations. The proposed FPGLBlock consists of two key components: the Fused Fourier Convolution Mixer (FFCM) and the Prior-Gated Feed Forward Network (PGFN).

Let the input feature representation of the i-th FPGLBlock be denoted as *F^i^*^−1^, with the corresponding prior mask represented as *M^i^*^−1^. The overall computation process of FPGLBlock can be formulated as:

(5) $${\tilde{F}}^{i}=F^{i-1}+\mathrm{FFCM}\left(\mathrm{BN}\left(F^{i-1}\right)\right)$$

(6) $$F^{i},M^{i}=\mathrm{PGFN}\left(\mathrm{BN}\left({\tilde{F}}^{i}\right),M^{i-1}\right)+{\tilde{F}}^{i}$$

where ${\tilde{F}}^{i}$ denotes the enhanced feature representation generated by FFCM, *F^i^* represents the output feature representation of the i-th FPGLBlock, and *M^i^* indicates the dynamic prior information updated by PGFN. Through residual learning, the proposed FPGLBlock effectively alleviates the gradient degradation problem during the training of deep networks while maintaining feature representation stability. It provides more robust high-level semantic representations for subsequent processing stages.

#### 3.3.1. FFCM

This paper proposes a Fusion Fourier Convolution Mixer (FFCM) module. Specifically, FFCM first employs multi-scale depthwise convolutions to extract local temporal patterns across different receptive fields. Subsequently, the extracted local features are projected into the frequency domain, where global dependencies are established through learnable frequency-domain transformations. Finally, local temporal information and global spectral interactions are jointly integrated, enabling efficient feature mixing for EEG representation learning.

Let the input feature map be denoted as $F\in R^{C\times 1\times T_{3}}$, where *C* represents the number of feature channels and *T*_3_ denotes the temporal dimension. The overall procedure of FFCM can be formulated as:

(7) $$F_{\mathrm{FFCM}}^{i}=\delta \left(\zeta \left(F^{i-1}\right)\right)$$

where *ζ*(⋅) denotes the local multi-scale feature extraction process, and *δ*(⋅) represents the global frequency-domain interaction operation.

##### Local Multi-Scale Temporal Feature Modeling

Emotion-related patterns in EEG signals usually exhibit variations across multiple temporal scales, like transient fluctuations and persistent rhythmic activities. A single-scale convolution operation is insufficient to simultaneously capture short-term variations and long-range temporal contexts. Therefore, FFCM introduces a multi-scale depthwise convolution strategy to construct local temporal feature representations.

First, a 1 × 1 point-wise convolution is applied to expand the channel dimension and perform feature transformation:

*F*_0_ = *PConv*(*F^i^*^−1^) (8)

where *PConv*(⋅) denotes the point-wise convolution operation. The expanded feature maps are subsequently divided into two feature groups along the channel dimension:

(9) $$F_{0}=\left[F_{1},F_{2}\right]\;F_{1},F_{2}\in R^{2C\times 1\times T_{3}}$$

Different depthwise convolutions with kernels of 1 × 3 and 1 × 5 are then applied to extract multi-scale temporal patterns. This design allows the FFCM to capture both short-range and relatively long-range local temporal dependencies at the feature level, and enables the network to model global EEG dynamics across different time scales before applying the Fourier domain. This process can be represented as:

*F_l_* = *ζ*(*F^i^*^−1^) = *Concat*(*ϕ*(*DConv*_1×3_(*F*_1_)),*ϕ*(*DConv*_1×5_(*F*_2_))) (10)

where *ϕ*(⋅) represents the GELU activation function, and DConv denotes depthwise convolution with different kernel sizes.

##### Global Frequency-Domain Interaction

Although convolution-based operations are effective in capturing local temporal variations, their limited receptive fields restrict the ability to model long-range dependencies. To overcome this limitation, FFCM introduces a Fourier-domain interaction mechanism, which transforms local feature representations into the frequency domain for global dependency modeling.

Given the local feature representation *F_l_*, a fast Fourier transform is first applied to project the feature map into the frequency domain:

(11) $$Z\left(u,v\right)=F\left(F_{l}\right)=\frac{1}{\sqrt{\mathrm{HL}}}\sum_{h=0}^{H-1}\sum_{l=0}^{L-1}F_{l}\left(h,l\right)e^{-j2\pi \left(\frac{\mathrm{hu}}{H}+\frac{\mathrm{lv}}{L}\right)}$$

where ℱ(⋅) denotes the Fourier transform. Since the frequency-domain representation contains complex-valued components, the real and imaginary parts are jointly encoded. Subsequently, the real and imaginary parts of the frequency domain are jointly mapped using a 1 × 1 convolution, thereby enabling cross-channel frequency domain feature interaction:

*F_g_* = ℱ^−1^(*σ*(*BN*(*Conv*_1×1_(*Z*)))) (12)

where ℱ^−1^(⋅) denotes the inverse Fourier transform, *BN*(⋅) represents batch normalization, and *σ*(⋅) indicates the nonlinear activation function.

#### 3.3.2. PGFN

Although FFCM can obtain comprehensive global feature representations through joint local-frequency modeling, the importance of EEG responses at different temporal locations is not consistent for emotion recognition. Therefore, a Prior-Gated Feed Forward Network (PGFN) is further proposed in this work, which utilizes dynamic prior information to adaptively modulate feature representations and highlight discriminative EEG patterns.

The proposed module adopts a dual-branch collaborative architecture. The feature branch is responsible for feature transformation and representation learning, where PConv and depthwise convolution are employed to capture local temporal information. Meanwhile, the prior branch takes the input prior mask as a basis and generates two groups of gating weights through depthwise convolutions with different kernel sizes, enabling adaptive regulation of multi-scale feature responses. Given the input feature representation $F_{\mathrm{FFCM}}^{i}\in R^{C\times 1\times T_{3}}$, the overall process of PGFN can be formulated as:

(13) $$\left(F_{\mathrm{PGFN}}^{i},M^{i}\right)=\psi \left(F_{\mathrm{FFCM}}^{i},M^{i-1}\right)$$

where *M^i^*^−1^ denotes the prior mask propagated from the previous layer, and *ψ*(⋅) represents the prior-gated feed-forward mapping.

(1) Feature Branch

First, PConv projects the input features into a higher-dimensional feature space, followed by depthwise convolution to further incorporate local contextual information. Subsequently, the transformed features are split along the channel dimension into two feature groups so that they can be individually modulated by different prior-guided gating weights. The overall process of the feature branch is formulated as:

(14) $$F_{3},F_{4}=\mathrm{split}\left(\phi \left(\mathrm{DConv}\left(\mathrm{PConv}\left(F_{\mathrm{FFCM}}^{i}\right)\right)\right)\right)$$

(2) Prior Branch

To construct the initial prior information, this work exploits the dynamic variation in feature responses along the channel dimension. Unlike conventional attention mechanisms, which typically learn an attention coefficient through a parameterized attention function, the proposed prior mask is generated from intrinsic feature variation and dynamically guides feature propagation. Specifically, the initial prior map is generated as follows:

(15) $$M^{0}=\underset{1\leq c\leq C}{\max}F_{\mathrm{FFCM}}^{i}-\underset{1\leq c\leq C}{\min}F_{\mathrm{FFCM}}^{i}$$

For each temporal position, the difference responses across all feature channels are calculated, producing a dynamic prior map. Compared with fixed weighting strategies, this approach allows the model to generate sample-dependent temporal priors, providing adaptive guidance for subsequent feature modulation. It is worth noting that this prior is not intended to directly encode a specific neurophysiological mechanism of emotion.

To further capture prior information at different temporal scales, convolution operations with different kernel sizes are applied to the prior mask for feature modeling and projection:

*M*_1_,*M*_2_ = *σ*(*Conv*_1×*k*_(*split*(*PConv*(*M^i^*^−1^)))) (16)

where *k* = {3,5}. Subsequently, the generated gating weights are applied to the two feature groups from the feature branch, enabling prior-guided dynamic feature selection. The process is formulated as:

*F_g_* = *Concat*(*M*_1_⨀*F*_3_,*M*_2_⨀*F*_4_) (17)

where ⊙ denotes element-wise multiplication, and *Concat*(⋅) represents channel-wise concatenation.

Finally, the fused features are projected back to the original channel dimension through convolution. Meanwhile, the two groups of gating weights are utilized to update the prior mask, and the refined prior information is propagated to the subsequent FPGLBlock. This cross-layer propagation mechanism enables continuous refinement of dynamic prior information. The process is formulated as:

(18) $$F_{\mathrm{PGFN}}^{i},M^{i}=\mathrm{PConv}\left(F_{g},\mathrm{Concat}\left(M_{1},M_{2}\right)\right)$$

### 3.4. Global Pooling

The output feature representation $Z_{n}\in R^{C\times 1\times T_{3}}$ from the stacked FPGLBlocks is fed into the Global Average Pooling (GAP) layer to compress the channel dimension and obtain the global feature vector:

*f* = *GAP*(*Z_n_*) ∈ *R*^1×^*^T^* (19)

The resulting feature vector is then flattened and passed through a fully connected layer to produce the final prediction output:

(20) $$\hat{y}=\mathrm{Softmax}\left(W_{f}\cdot \mathrm{Flatten}\left(f\right)+b\right)$$

where *W_f_* and *b* denote trainable parameters, and $\hat{y}$ represents the probability distribution over all emotion categories.

## 4. Experiments and Results

This section aims to experimentally evaluate and analyse the proposed FPGLNet model. Comprehensive experiments are conducted on two publicly available EEG datasets. To provide a thorough assessment of model performance, FPGLNet is compared with several representative EEG emotion recognition approaches, including conventional machine learning methods and advanced deep neural network-based models. Furthermore, we conduct ablation experiments to systematically verify the contributions of each key component and the influence of network depth on overall performance.

### 4.1. Datasets

In this study, extensive experiments are conducted on two publicly available EEG emotion recognition datasets, SEED-IV (four-class classification) and SEED-V (five-class classification). Both datasets are collected by the BCMI Laboratory of Shanghai Jiao Tong University and involve different experimental protocols and emotion categories, providing a comprehensive evaluation of model performance across different emotion categories, sessions, and subjects.

SEED-IV [22] is an extended version of the original SEED dataset, where the emotion categories are expanded from three classes to four classes: happy, sad, fear, and neutral. The dataset consists of 15 subjects (6 males and 9 females). During each session, participants were instructed to watch 24 emotion-inducing video clips, with six clips corresponding to each emotion category. Each video lasted approximately two minutes. EEG signals were recorded using 62 standard electrode channels at a sampling rate of 200 Hz. A band-pass filter ranging from 4 to 47 Hz was applied to remove low-frequency drift and high-frequency interference. In addition, artifact removal procedures were performed to eliminate ocular artifacts (EOG) and muscle interference (EMG), thereby improving signal quality.

SEED-V [23] further extends SEED-IV by increasing the number of emotion categories to five: happy, sad, fear, disgust, and neutral. The dataset contains recordings from 16 subjects, where each subject participated in multiple experimental sessions. The duration and experimental design of the video stimuli are consistent with those in SEED-IV, with each video lasting approximately two minutes. The number of video clips was balanced across emotion categories to maintain a relatively uniform distribution of samples among different classes. In addition, EEG signals were acquired using the same electrode configuration and sampling frequency as SEED-IV.

In the experimental setup, the continuous EEG recordings were segmented into independent samples using non-overlapping 2 s windows. Each sample preserved the original channel arrangement and temporal sequence, which were subsequently used for feature learning and model training. After preprocessing, all EEG segments obtained from different subjects and sessions were pooled together and randomly shuffled at the segment level. The resulting samples were then divided into training and testing sets at a ratio of 4:1, with 80% used for training and the remaining 20% used for testing. Both datasets were partitioned using the above method, and the input configurations and sample statistics are summarized in Table 1.

### 4.2. Feature Dimension Analysis

To provide an intuitive illustration of the feature evolution throughout different stages of the proposed network, the input and output dimensions of the main modules are summarized in Table 2. This analysis facilitates the investigation of feature transformation processes across the temporal domain, spatial domain, and high-level representation stages.

As shown in Table 2, the original EEG input has a size of 1 × 62 × 400. After the temporal convolution operation, the temporal dimension is reduced from 400 to 376, while the number of feature channels is expanded to 64, enabling the extraction of temporal patterns from the original EEG sequence. Subsequently, the spatial convolution integrates information across electrode channels, reducing the spatial dimension from 62 to 1 and achieving spatial feature aggregation.

Afterwards, the average pooling operation further reduces the temporal dimension from 376 to 21, effectively shortening the subsequent sequence length and improving computational efficiency. The FPGLBlocks are stacked N times, where the input and output feature dimensions remain unchanged at 64 × 1 × 21. By maintaining consistent feature dimensions during deep feature learning, the proposed architecture avoids additional dimensional transformations and the associated parameter increase. This design not only reduces redundant parameters and computational costs but also facilitates stable feature propagation, leading to improved computational efficiency.

Finally, global average pooling and the fully connected layer are employed to perform feature compression and category prediction. Overall, the proposed network progressively compresses temporal and spatial information during the front-end convolutional encoding stage. Before entering the stacked FPGLBlocks, the sequence length is significantly reduced, making the subsequent feature interaction process more computationally manageable. The gradual and well-organized feature dimension transition contributes to maintaining both representation capability and training stability.

### 4.3. Results Analysis

To evaluate the overall performance of the proposed FPGLNet, comprehensive experiments are conducted on the SEED-IV and SEED-V emotion classification datasets. The evaluation focuses on three aspects: the convergence behavior during the training process, the final classification accuracy, and the per-class discriminative performance, including the confusion distribution among different emotion categories.

**Figure 3:**
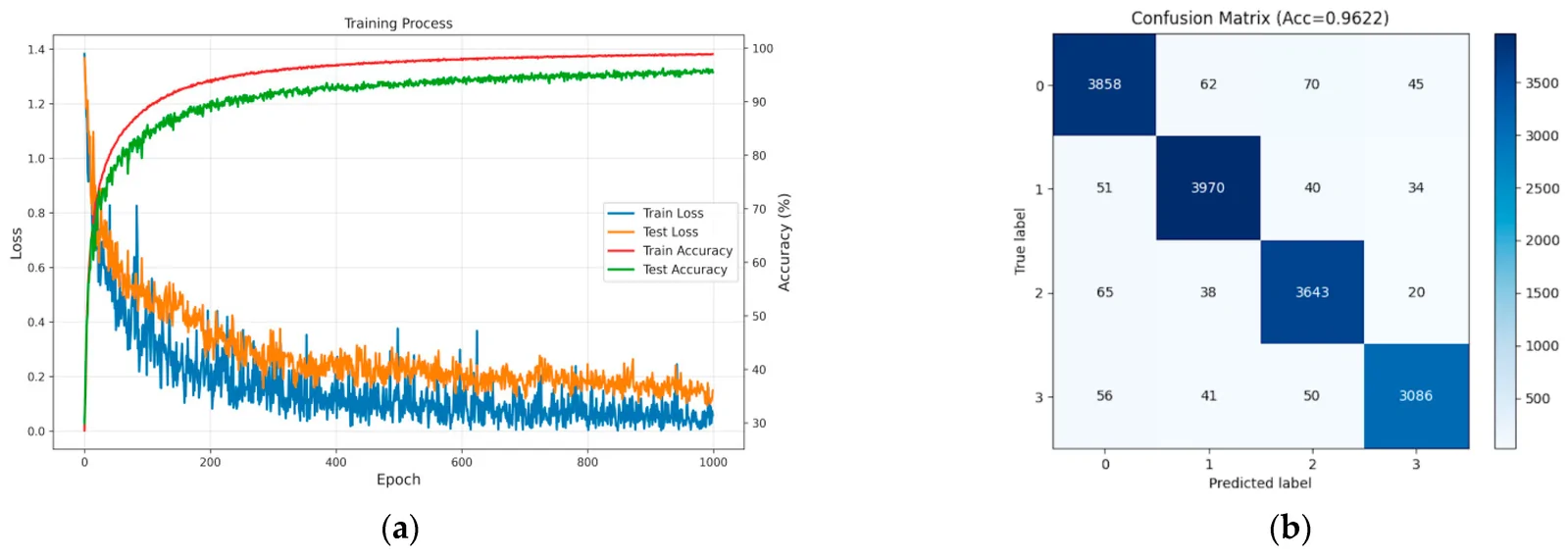
Experimental results on the SEED-IV dataset. (**a**) Training and testing process curves. (**b**) Confusion matrices obtained on the dataset.

As shown in Figure 3 and Table 3, the experimental results on the SEED-IV dataset demonstrate that FPGLNet achieves stable training behavior and rapid convergence in the four-class classification task, reaching a classification accuracy of 96.22%. Table 3 further reports the per-class metrics, where all four emotion categories achieve F1-scores above 95% (95.67–96.76%), with average precision, recall, specificity, and F1-score of 96.25%, 96.19%, 98.73%, and 96.21%, respectively. The confusion matrix reveals clear separability among different emotion categories, with only a small proportion of samples being misclassified. This indicates that the proposed model exhibits strong discriminative capability in characterizing complex emotional patterns contained in EEG signals.

**Figure 4:**
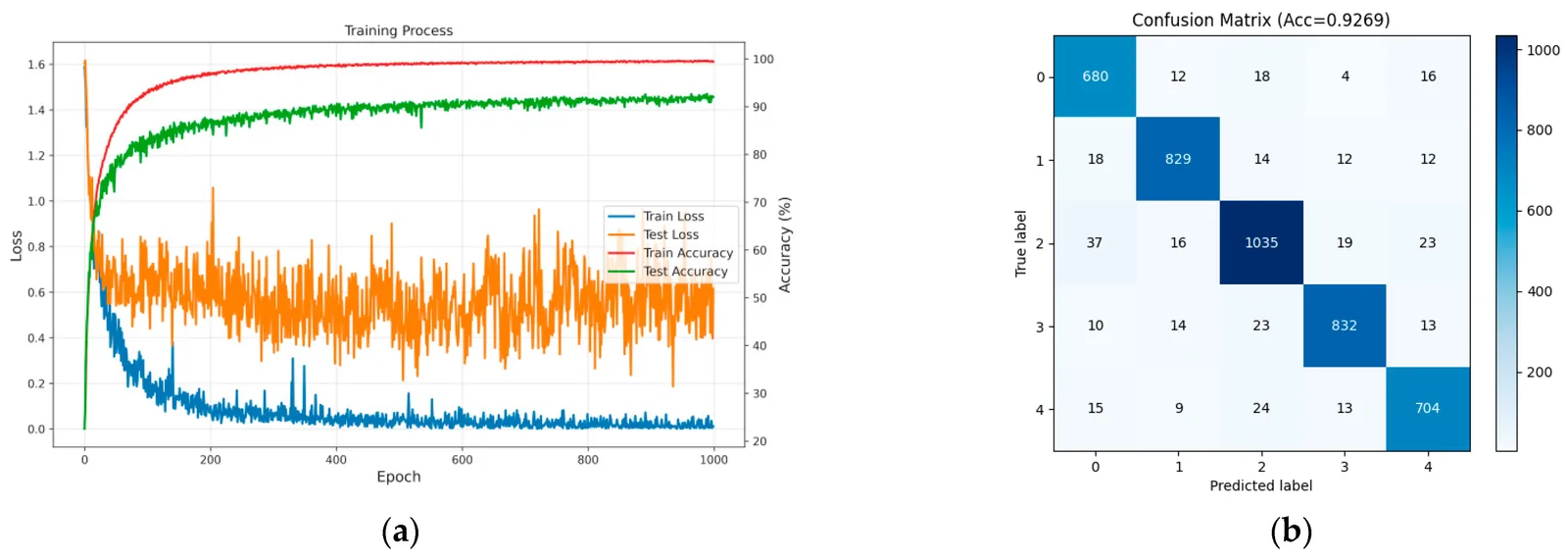
Experimental results on the SEED-V dataset. (**a**) Training and testing process curves. (**b**) Confusion matrices obtained on the dataset.

For the five-class classification task on the SEED-V dataset (Figure 4 and Table 4), FPGLNet continues to achieve high recognition performance with an accuracy of 92.69%, while maintaining a stable optimization trend during training. Although the increase in the number of emotion categories introduces greater classification difficulty, the proposed model still maintains relatively low confusion levels among multiple emotion categories. These results further verify the robustness and generalization capability of the proposed architecture in multi-emotion recognition scenarios.

### 4.4. Comparison with Some Methods

To comprehensively evaluate the effectiveness of the proposed FPGLNet, several representative EEG emotion recognition approaches published in recent years are selected for comparison. These methods include graph neural network-based approaches (RGNN, MDGCN-SRCNN), Transformer-based architectures (AMDET, EeT, CIT-EmotionNet, STAFNet), and lightweight models (FMAL, MSA). The comparison results are presented in Table 5.

As shown in Table 5, FPGLNet achieves a classification accuracy of 96.22% on the SEED-IV four-class classification task, outperforming all compared methods. Compared with the classical graph neural network models RGNN and MDGCN-SRCNN, the proposed method achieves accuracy improvements of 16.85 and 10.70 percentage points, respectively. Compared with Transformer-based approaches AMDET, EeT, CIT-EmotionNet, and STAFNet, FPGLNet achieves improvements of 8.90, 12.95, 4.03 and 2.58 percentage points, respectively.

Furthermore, compared with lightweight models based on frequency-domain modeling, including FMAL and MSA, FPGLNet achieves accuracy improvements of 4.55 and 0.32 percentage points, respectively. Regarding model complexity, FPGLNet contains only 0.705 M parameters with a computational cost of 0.153 GMACs. Although its parameter size is slightly larger than that of lightweight approaches such as FMAL (0.517 M), it remains substantially more compact than most Transformer-based and graph neural network-based models.

Overall, FPGLNet delivers the best classification performance among the compared approaches while maintaining low computational complexity, demonstrating the effectiveness of the proposed architecture for EEG emotion recognition tasks.

To further evaluate the effectiveness of the proposed FPGLNet in complex emotion recognition scenarios, comparative experiments are conducted on the SEED-V five-class EEG emotion recognition dataset against several representative methods in recent years, including AP-CapsNet, ASTDF-Net, CNN, CLIER, and MSA. The comparison results are presented in Table 6.

As shown in Table 6, FPGLNet achieves an accuracy of 92.69% on the SEED-V dataset with only 0.928 M parameters and 0.163 G MACs, obtaining the best performance among all compared methods. Compared with AP-CapsNet, ASTDF-Net, CNN, and CLIER, the proposed method improves the classification accuracy by 11.90, 9.06, 10.63, and 10.23 percentage points, respectively. Moreover, compared with the competitive lightweight model MSA, FPGLNet achieves a performance improvement of 6.77 percentage points while substantially reducing the number of parameters and computational cost. These results demonstrate that the proposed network possesses stronger discriminative capability and more effective feature representation ability for complex multi-class emotion recognition tasks.

The SEED-V dataset contains five emotion categories, where the boundaries between different emotional classes are relatively ambiguous. EEG signals also exhibit higher inter-class similarity and intra-class variability, imposing greater demands on feature modeling capability. To address these challenges, the proposed Fused Fourier Convolution Mixer (FFCM) effectively captures global frequency-domain information and local spatiotemporal patterns in EEG signals, thereby enhancing discriminative feature representations across different emotional states. Meanwhile, the Prior-Gated Feed Forward Network (PGFN) employs a prior-guided gating mechanism to dynamically select and recalibrate emotion-related features, further strengthening informative patterns from critical brain regions and important temporal segments while suppressing noise and redundant information. Through the complementary interaction between FFCM and PGFN, FPGLNet is capable of characterizing complex emotional patterns more effectively, leading to improved recognition performance on the five-class classification task.

Overall, the experimental results demonstrate that the proposed method achieves significant performance improvements while maintaining a lightweight architecture, highlighting its computational efficiency, practical applicability, and strong representation capability for EEG emotion patterns.

### 4.5. Ablation Study

#### 4.5.1. Impact of FFCM and PGFN on Model Performance

To systematically evaluate the contributions of Fused Fourier Conv Mixer (FFCM) and Prior-Gated Feed Forward Network (PGFN) to the overall performance of FPGLNet, ablation experiments are conducted on the SEED-IV and SEED-V datasets. Four variants are constructed for comparison: the baseline model without both FFCM and PGFN (NoFPGL), the model equipped only with FFCM, the model equipped only with PGFN, and the complete FPGLNet incorporating both modules. The classification performance of different architectures is compared, and the results are presented in Table 7.

The experimental results demonstrate that the baseline model without FFCM and PGFN achieves the lowest classification performance on both datasets, obtaining accuracies of only 72.17% and 72.72% on SEED-IV and SEED-V, respectively. Due to the strong non-stationarity and low signal-to-noise ratio characteristics of EEG signals, relying solely on the basic network components, such as PatchEmbedding and the classification head, is insufficient to fully capture discriminative emotion-related features, resulting in limited recognition capability.

After introducing FFCM, the classification performance is significantly improved, achieving accuracies of 94.3% and 89.77% on the SEED-IV four-class and SEED-V five-class emotion recognition tasks, respectively. Meanwhile, the model equipped only with PGFN also achieves considerable improvements over the baseline, reaching accuracies of 87.26% and 78.65% on SEED-IV and SEED-V, respectively.

Furthermore, when both FFCM and PGFN are integrated into the complete FPGLNet, the model achieves the best classification performance among these four variants. Compared with models containing only a single module, the complete architecture obtains further improvements on both datasets, achieving the optimal accuracies of 96.22% on SEED-IV and 92.69% on SEED-V, demonstrating the complementary effects of the two modules. Specifically, FFCM enhances the multi-scale frequency representation capability of EEG signals by jointly modeling global Fourier frequency information and local convolutional features, while PGFN generates dynamic gating weights based on residual contextual priors to emphasize emotion-related discriminative EEG features and suppress noise and redundant information.

These results indicate that the collaborative interaction between FFCM and PGFN enables the model to learn more comprehensive emotion-discriminative representations from EEG signals. Notably, while maintaining a relatively low number of parameters and computational complexity, FPGLNet continues to achieve performance improvements, indicating that the proposed lightweight architecture can effectively enhance emotional feature learning with limited computational overhead. This further validates the effectiveness of the proposed lightweight network design.

#### 4.5.2. The Impact of Different FPGLBlock Numbers on Model Performance

**Figure 5:**
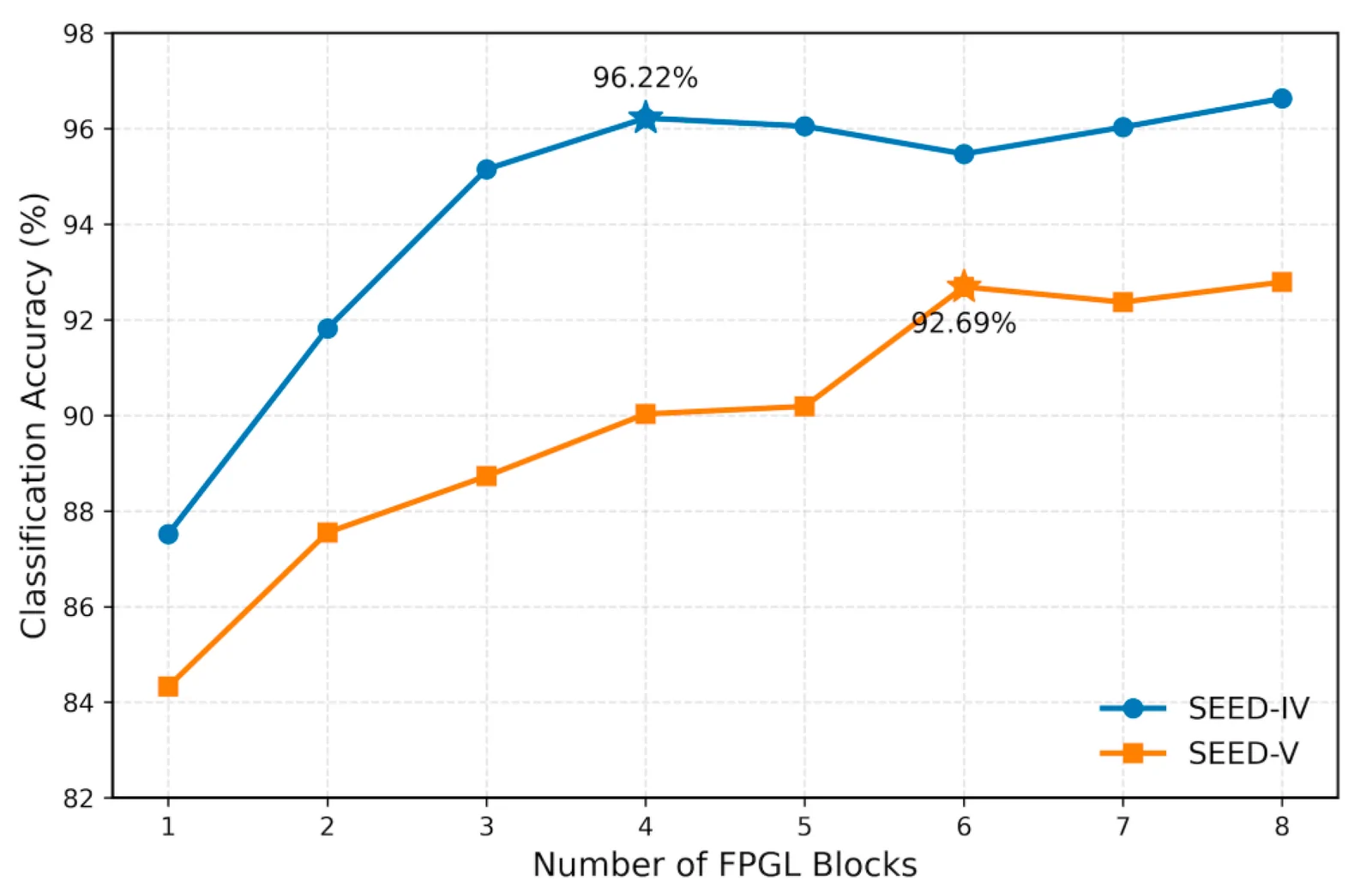
Experimental results with different numbers of FPGLBlocks.

To further analyse the influence of network depth on model performance and validate the effectiveness of the proposed FPGLBlock, we conduct depth ablation experiments on both the SEED-IV and SEED-V datasets after finishing the main experiments. By varying the number of stacked FPGLBlocks from 1 to 8, the classification performance under different network depths is systematically evaluated while keeping the overall network architecture and training strategy unchanged. The experimental results are illustrated in Figure 5.

As shown in Figure 5, increasing the number of FPGLBlocks leads to a gradual improvement in classification performance on both datasets, followed by a tendency toward saturation. This indicates that an appropriate increase in network depth plays an important role in modeling EEG emotion-related features. When only a small number of FPGLBlocks are employed, the shallow feature transformation limits the ability of the model to sufficiently capture complex spatiotemporal dependencies and frequency coupling characteristics in EEG signals, resulting in relatively lower classification performance. All data points plotted in Figure 5 are test-set accuracies of independently trained models with different block counts.

However, with the continuous increase in the number of FPGLBlocks, the overall performance gradually enters a saturation stage. On the SEED-IV dataset, the model achieves a classification accuracy of 96.22% with four FPGLBlocks. Further increasing the network depth brings slight performance fluctuations, with the accuracy variation below 0.5%. For the SEED-V dataset, models with 6–8 FPGLBlocks maintain comparable recognition performance. Due to the inherent randomness and non-stationarity of EEG signals, excessively deep architectures may introduce redundant feature representations, causing the effective information gain from additional blocks to gradually diminish and eventually resulting in performance saturation or degradation.

It is worth noting that FPGLNet is designed for lightweight EEG emotion recognition and edge-device deployment. Therefore, the selection of network depth should consider not only classification performance but also model parameters, computational complexity, and inference efficiency. As the number of FPGLBlocks increases, the model parameters and computational cost exhibit an approximately linear growth trend, while the improvement in classification accuracy gradually becomes saturated, showing an evident diminishing marginal benefit. In our main experiments, we preset four FPGLBlocks for SEED-IV and six FPGLBlocks for SEED-V before training. This ablation further demonstrates that this preset configuration achieves a good balance between classification performance and model overhead.

In summary, the preset number of FPGLBlocks achieves a favorable balance among feature representation capability, model complexity, and generalization performance. The ablation results further validate the effectiveness of the proposed FPGLNet architecture. These findings demonstrate that FPGLNet can achieve comprehensive EEG feature modeling while maintaining low parameter complexity and computational cost, providing an effective solution for lightweight EEG emotion recognition model design.

## 5. Conclusions

This paper proposes FPGLNet, which performs global frequency-domain modeling through FFCM and achieves prior-guided dynamic feature selection through PGFN. By integrating frequency-aware representation learning with adaptive feature modulation, the proposed network improves EEG emotion recognition performance while maintaining a lightweight architecture. Experimental results demonstrate that FPGLNet achieves high classification accuracy on both the SEED-IV and SEED-V datasets, while exhibiting favorable advantages in terms of parameter size and computational complexity. These results validate the effectiveness of the proposed network design.

Although the proposed method achieves promising performance, several aspects remain worthy of further investigation. All experiments are conducted on publicly collected data under laboratory-controlled environments, and the model performance may degrade when facing real-world EEG data with strong artifacts and electrode interference. Cross-subject generalization still needs further enhancement.

Future research will focus on the following directions: (1) exploring domain generalization capability under cross-subject and cross-dataset scenarios to further improve model robustness in complex application environments; (2) integrating knowledge distillation, network quantization, and neural architecture search techniques to further reduce computational overhead and meet the requirements of real-time edge deployment; (3) extending the proposed method to multimodal emotion recognition and online brain–computer interface systems to evaluate its stability and practical applicability in real-world scenarios.

## Figures and Tables

**Table 1:** SEED-IV and SEED-V dataset sample distribution.

Dataset	Data Shape	Total Number of Samples	Number of Training Samples	Number of Test Samples
SEED-IV	62 × 400	75,645	60,516	15,129
SEED-V	62 × 400	22,008	17,606	4402

**Table 2:** Input and output dimensions of the main modules of the model. The arrow indicates that the fully connected layer maps the 64-dimensional features to the target class outputs.

Module	Input	Output	Parameters
Time CNN	1 × 62 × 400	64 × 62 × 376	1664
Spatial CNN	64 × 62 × 376	64 × 1 × 376	254,016
BatchNorm + ELU	64 × 1 × 376	64 × 1 × 376	128
AvgPool2d	64 × 1 × 376	64 × 1 × 21	0
Liner + ELU	64 × 1 × 21	64 × 1 × 128	2816
FPGLBlock × 4	64 × 1 × 128	64 × 1 × 128	447,060
GlobalAvgPool2d	64 × 1 × 128	64 × 1 × 1	0
Global Pooling FC (64 → class)	64	4	260

**Table 3:** Comprehensive classification performance of FPGLNet on the SEED-IV.

Class	Precision (%)	Recall/Sensitivity (%)	Specificity (%)	F1-Score (%)
0/fear	95.73	95.61	98.45	95.67
1/sad	96.57	96.95	98.72	96.76
2/neutral	95.79	96.73	98.59	96.26
3/happy	96.89	95.45	99.17	96.17
Avg	96.25	96.19	98.73	96.21

**Table 4:** Comprehensive classification performance of FPGLNet on the SEED-V.

Class	Precision (%)	Recall/Sensitivity (%)	Specificity (%)	F1-Score (%)
0-disgust	89.47	93.15	97.82	91.28
1-fear	94.20	93.67	98.55	93.94
2-sad	92.91	91.59	97.59	92.25
3-neutral	94.55	93.27	98.63	93.91
4-happy	91.67	92.03	98.24	91.85
Avg	92.56	92.74	98.17	92.65

**Table 5:** Comparison of experimental results on the SEED-IV dataset.

Year	Method	Accuracy (%)	Params (M)	MACs (G)
2020	RGNN [24]	79.37	-	-
2022	MDGCN-SRCNN [25]	85.52	-	-
2022	AMDET [26]	87.32	-	
2022	EeT [27]	83.27	-	-
2024	CIT-EmotionNet [28]	92.09	-	-
2024	STAFNet [29]	93.64	-	-
2026	MSA [30]	95.90	1.717	0.470
2026	FMAL [31]	91.67	0.517	0.159
-	FPGLNet	96.22	0.705	0.153

**Table 6:** Comparison of experimental results on the SEED-V dataset.

Year	Method	Accuracy (%)	Params (M)	MACs (G)
2023	AP-CapsNet [32]	80.79	-	-
2023	ASTDF-Net [33]	83.63	-	-
2024	CNN [34]	82.06	-	-
2025	CLIER [35]	82.46	-	-
2026	MSA [30]	85.92	1.717	0.470
	FPGLNet	92.69	0.928	0.163

**Table 7:** Ablation study results of FFCM and PGFN modules.

Dataset	No FPGL	FFCM	PGFN	FPGL
SEED-IV	72.17	94.30	87.26	96.22
SEED-V	72.72	89.77	78.65	92.69

## Data Availability

The original contributions presented in this study are included in the article. Further inquiries can be directed to the corresponding author.

## References

[1] Dolan R.J. (2002). Emotion, cognition, and behavior. Science.

[2] Frantzidis C.A., Bratsas C., Papadelis C.L., Konstantinidis E., Pappas C., Bamidis P.D. (2010). Toward emotion-aware computing: An integrated approach using multichannel neurophysiological recordings and affective visual stimuli. IEEE Trans. Inf. Technol. Biomed..

[3] Zhang T., Zheng W., Cui Z., Zong Y., Yan J., Yan K. (2016). A deep neural network-driven feature learning method for multi-view facial expression recognition. IEEE Trans. Multimed..

[4] Liu Z.T., Xie Q., Wu M., Cao W.-H., Mei Y., Mao J.-W. (2018). Speech emotion recognition based on an improved brain emotion learning model. Neurocomputing.

[5] Britton J.C., Phan K.L., Taylor S.F., Welsh R.C., Berridge K.C., Liberzon I. (2006). Neural correlates of social and nonsocial emotions: An fMRI study. NeuroImage.

[6] Zhang T., Wang X., Xu X., Chen C.L.P. (2022). GCB-net: Graph convolutional broad network and its application in emotion recognition. IEEE Trans. Affect. Comput..

[7] Zheng W.L., Zhu J.Y., Lu B.L. (2019). Identifying stable patterns over time for emotion recognition from EEG. IEEE Trans. Affect. Comput..

[8] Hjorth B. (1970). EEG analysis based on time domain properties. Electroencephalogr. Clin. Neurophysiol..

[9] Liu Y., Sourina O. (2013). Real-time fractal-based valence level recognition from EEG. Transactions on Computational Science XVIII.

[10] Aftanas L.I., Reva N.V., Varlamov A.A., Pavlov S.V., Makhnev V.P. (2004). Analysis of evoked EEG synchronization and desynchronization in conditions of emotional activation in humans: Temporal and topographic characteristics. Neurosci. Behav. Physiol..

[11] Sanei S., Chambers J.A. (2007). EEG Signal Processing.

[12] Shi L.C., Jiao Y.Y., Lu B.L. Differential entropy feature for EEG-based vigilance estimation. Proceedings of the 35th Annual International Conference of the IEEE Engineering in Medicine and Biology Society (EMBC).

[13] Zheng W.L., Zhu J.Y., Peng Y., Lu B.-L. EEG-based emotion classification using deep belief networks. Proceedings of the IEEE International Conference on Multimedia and Expo (ICME).

[14] Liu J., Feng N., Liang Y. (2025). Frequency-domain hybrid model for EEG-based emotion recognition. Mathematics.

[15] Zhu Z., Jin Z., Wang G., Zeng X., Zeng F. (2025). Research progress in emotion recognition based on EEG monitoring technology. Chin. J. Sens. Actuators.

[16] Liu Z., Lin Y., Cao Y., Hu H., Wei Y., Zhang Z., Lin S., Guo B. Swin transformer: Hierarchical vision transformer using shifted windows. Proceedings of the IEEE/CVF International Conference on Computer Vision (ICCV).

[17] Lu Y., Yao X. (2024). A review of emotion recognition based on EEG signal analysis. Fudan Univ. J. Nat. Sci..

[18] Chen J., Ran X. (2019). Deep learning with edge computing: A review. Proc. IEEE.

[19] Shi W., Cao J., Zhang Q., Li Y., Xu L. (2016). Edge computing: Vision and challenges. IEEE Internet Things J..

[20] Hinton G., Vinyals O., Dean J. Distilling the knowledge in a neural network. Proceedings of the NIPS Deep Learning and Representation Learning Workshop.

[21] Han S., Mao H., Dally W.J. Deep compression: Compressing deep neural networks with pruning, trained quantization and Huffman coding. Proceedings of the 14th International Conference on Learning Representations (ICLR).

[22] Ye W., Zhang Z., Teng F., Zhang M., Wang J., Ni D., Li F., Xu P., Liang Z. (2025). Semi-supervised dual-stream self-attentive adversarial graph contrastive learning for cross-subject EEG-based emotion recognition. IEEE Trans. Affect. Comput..

[23] Li Y., Zheng W., Cui Z., Zhang T., Zong Y. A novel neural network model based on cerebral hemispheric asymmetry for EEG emotion recognition. Proceedings of the 27th International Joint Conference on Artificial Intelligence (IJCAI).

[24] Zhong P., Wang D., Miao C. (2022). EEG-based emotion recognition using regularized graph neural networks. IEEE Trans. Affect. Comput..

[25] Bao G., Yang K., Tong L., Shu J., Zhang R., Wang L., Yan B., Zeng Y. (2022). Linking Multi-Layer DynamicalGCN with Style-Based Recalibration CNN for EEG-Based Emotion Recognition. Front. Neurorobot..

[26] Xu Y., Du Y., Li L., Lai H., Zou J., Zhou T., Xiao L., Liu L., Ma P. (2024). AMDET: Attention-based multiple dimensions EEG transformer for emotion recognition. IEEE Trans. Affect. Comput..

[27] Liu J., Wu H., Zhang L., Zhao Y. Spatial-temporal transformers for EEG emotion recognition. Proceedings of the 6th International Conference on Advances in Artificial Intelligence (ICAAI).

[28] Lu W., Xia L., Tan T.P., Ma H. (2024). CIT-EmotionNet: Convolution interactive transformer network for EEG emotion recognition. PeerJ Comput. Sci..

[29] Hu F., He K., Qian M., Liu X., Qiao Z., Zhang L., Xiong J. (2024). STAFNet: An adaptive multi-feature learning network via spatiotemporal fusion for EEG-based emotion recognition. Front. Neurosci..

[30] Liu S., Bi Y., Huo L., Gan Q., Zhou S. (2026). EEG emotion recognition model integrating a memory-forgetting mechanism and self-attention. J. Electron. Inf. Technol..

[31] Bi Y., Yuan Y., Liu S., Huo L. (2026). A lightweight EEG emotion recognition model based on multi-electrode channel aggregation. J. Sichuan Univ. Nat. Sci. Ed..

[32] Liu S., Wang Z., An Y., Zhao J., Zhao Y., Zhang Y.-D. (2023). EEG emotion recognition based on the attention mechanism and pre-trained convolution capsule network. Knowl. Based Syst..

[33] Gong P., Jia Z., Wang P., Zhou Y., Zhang D. ASTDF-Net: Attention-based spatial-temporal dual-stream fusion network for EEG-based emotion recognition. Proceedings of the 31st ACM International Conference on Multimedia (MM).

[34] Aslan M., Baykara M., Alakus T.B. (2024). Analysis of brain areas in emotion recognition from EEG signals with deep learning methods. Multimed. Tools Appl..

[35] Li D., Jin Z., Shen Y., Wang Z., Jiang S. (2025). EEG emotion recognition based on an implicit emotion regulatory mechanism. IEEE Trans. Artif. Intell..

